# Retraction: Zheng L. *et al*. An Epidemiological Study of Risk Factors of Thyroid Nodule and Goiter in Chinese Women. *Int. J. Environ. Res. Public Health* 2015, *12*, 11608–11620

**DOI:** 10.3390/ijerph121114114

**Published:** 2015-11-04

**Authors:** International Journal of Environmental Research and Public Health Editorial Office

**Affiliations:** MDPI AG, Klybeckstrasse 64, 4057 Basel, Switzerland; E-Mail: ijerph@mdpi.com

At the request of the authors, the published article [1] will be retracted. The manuscript is a duplicate of another publication by the same authors [2]. The paper was submitted to the other journal two months earlier than to the *International Journal of Environmental Research and Public Health* (*IJERPH*) and was published online nearly one month after the submission to *IJERPH*. Our policy is to consider only original, unpublished articles that are not under review by any other journal, and all authors explicitly agree to this as a condition for processing their submissions. The authors have stated that the duplicate submission was caused by a lack of internal communication, for which they deeply apologize.

MDPI is a member of the Committee on Publication Ethics and takes the responsibility to enforce strict ethical policies and standards very seriously. To ensure the addition of only high quality scientific works to the field of scholarly publication, paper [1] is retracted and shall be marked accordingly. We apologize to our readership for any inconvenience caused.

## Supplementary Files

Supplementary File 1
